# Immune checkpoint inhibitor (nivolumab)-associated kidney injury and the importance of recognizing concomitant medications known to cause acute tubulointerstitial nephritis: a case report

**DOI:** 10.1186/s12882-018-0848-y

**Published:** 2018-02-27

**Authors:** Ryo Koda, Hirofumi Watanabe, Masafumi Tsuchida, Noriaki Iino, Kazuo Suzuki, Go Hasegawa, Naofumi Imai, Ichiei Narita

**Affiliations:** 1 Department of Nephrology, Uonuma Institute of Community Medicine, Niigata University Medical and Dental Hospital, 4132 Urasa, Minami Uonuma-shi, Niigata, 949-7302 Japan; 2 Department of Respiratory Medicine, Uonuma Institute of Community Medicine, Niigata University Medical and Dental Hospital, 4132 Urasa, Minami Uonuma-shi, Niigata, 949-7302 Japan; 3 Department of Pathology, Uonuma Institute of Community Medicine, Niigata University Medical and Dental Hospital, 4132 Urasa, Minami Uonuma-shi, Niigata, 949-7302 Japan; 4 Division of Clinical Nephrology and Rheumatology, Niigata University Graduate School of Medical and Dental Sciences, 1-757 Asahi-machi, Chuo-ku, Niigata, 951-8510 Japan

**Keywords:** Acute tubulointerstitial nephritis, Drug-induced lymphocyte stimulating test, Immune checkpoint inhibitor, Lansoprazole, Nivolumab, Proton pump inhibitor

## Abstract

**Background:**

Acute tubulointerstitial nephritis (ATIN) has been increasingly recognized as an important manifestation of kidney injury associated with the use of immune checkpoint inhibitors (anti-PD-1 and anti-CTLA-4). While the exact pathophysiology remains unknown, corticosteroids are the mainstay of management.

**Case presentation:**

We describe a 67-year-old man with stage IV non-small-cell lung cancer who developed kidney injury during treatment with the anti-PD-1 antibody nivolumab. A kidney biopsy showed ATIN without granuloma formation. Considering their mechanism of action, immune checkpoint inhibitors can alter immunological tolerance to concomitant drugs that have been safely used for a long time. For more than 4 years before the initiation of nivolumab therapy, the patient had been receiving the proton pump inhibitor lansoprazole, known to cause drug-induced ATIN, without significant adverse events including kidney injury. He showed rapid improvement in kidney function in 3 days (creatinine decreased from 2.74 to 1.82 mg/dl) on discontinuation of lansoprazole. He then received 500 mg intravenous methylprednisolone for 3 days followed by 1 mg/kg/day oral prednisolone and his creatinine levels eventually stabilized around 1.7 mg/dl. Drug-induced lymphocyte stimulation test (DLST) for lansoprazole was positive.

**Conclusions:**

The rapid improvement of kidney function after discontinuation and DLST positivity indicate that lansoprazole contributed to the development of ATIN during nivolumab therapy. Considering the time course, it is plausible that nivolumab altered the long-lasting immunological tolerance against lansoprazole in this patient. To the best of our knowledge, this is the first case report of DLST positivity for a drug that had been used safely before the initiation of an immune checkpoint inhibitor. Although corticosteroid therapy is recommended, the recognition and discontinuation of concomitant drugs, especially those known to induce ATIN, is necessary for the management of kidney injury associated with anti-PD-1 therapy.

## Background

Programmed cell death protein 1 (PD-1) and cytotoxic T-lymphocyte antigen 4 (CTLA-4) play a crucial role in anti-cancer immunity. Inhibitory signals to the host immune system produced by the interaction between PD-1 ligands (PD-L1 or PD-L2) expressed on cancer cells and PD-1 on T-lymphocytes allow cancer cells to avoid host immune surveillance [1]. CTLA-4 competes for binding with the B7 (CD80/86) molecule on antigen-presenting cells with CD28 on T-lymphocytes, provoking inhibitory signals to avoid excessive immunological response. The inhibition of these immune checkpoint proteins re-establish the response of T-lymphocytes against cancer cells. Monoclonal antibodies against PD-1 (nivolumab and pembrolizumab), PD-L1 (atezolizumab), and CTLA-4 (ipilimumab) are currently used for the treatment of advanced stage cancers and have demonstrated notable clinical efficacy [2]. However, the development of unique adverse effects called immune-related adverse events (irAEs) have been recognized during treatment with these immune checkpoint inhibitors (ICPIs) [3].

Although kidney involvement is relatively rare compared to other organs such as the skin, gastrointestinal tract, endocrine glands, and liver, at least two different types of ICPI-associated kidney injury (acute tubulointerstitial nephritis [ATIN] and immune complex mediated glomerulonephritis) have been reported [4]. ATIN seems to be the common histological feature of renal complications in irAEs [5, 6], but the reason for this remains unknown. Although the administration of corticosteroids based on the severity of kidney injury is recommended (0.5 to 1.0 mg/kg/day prednisone equivalents for Grade 2 or Grade 3, and 1.0 to 2.0 mg/kg/day for Grade 4 increased serum creatinine level) [4], no established strategy exists for the management of ICPI-associated ATIN.

Drug-specific T-cells have been shown to contribute to the development of drug-induced ATIN [7]; thus, the re-activation of the T-lymphocyte immune response by ICPI therapy may disrupt immunological tolerance to drugs that have been safely used previously, leading to the development of drug-induced ATIN.

Herein, we describe a patient with advanced stage lung cancer who developed kidney injury during treatment with the anti-PD-1 antibody nivolumab. The discontinuation of the proton pump inhibitor (PPI) lansoprazole, which is known to cause ATIN, resulted in the rapid improvement of kidney function before the initiation of steroid therapy. Drug-induced lymphocyte stimulation test (DLST) for lansoprazole was positive.

## Case presentation

A 67-year-old Japanese man was diagnosed with stage IV non-small-cell lung cancer with malignant pleural effusion in June 2015. His kidney function was normal at that time (creatinine 0.78 mg/dl, estimated glomerular filtration rate 76.2 ml/min/1.73 m^2^). His medical history included atrial fibrillation and gastroesophageal reflux disease. He had no known history of food or drug allergy. He did not drink but had smoked 40 cigarettes per day since at the age of 20 years. His family history was unremarkable. Despite receiving first-line chemotherapy (bevacizumab combined with pemetrexed plus cisplatin followed by maintenance pemetrexed infusion), the lung cancer progressed. Thus, nivolumab was initiated at a dose of 3 mg/kg every 2 weeks in April 2016. His baseline serum creatinine level before nivolumab therapy was 1.2 mg/dl, and red or white blood cells were not noted in urinalysis. Owing to general fatigue and appetite loss, 10 mg oral prednisolone daily was initiated in July 2016. Four months later, when the oral prednisolone treatment was tapered to 5 mg daily, sterile pyuria was noted and the creatinine level increased to 1.64 mg/dl. Since his creatinine level continued to increase, nivolumab was discontinued (a total of 19 cycles had been performed), and the patient was referred to the nephrology department in January 2017.

**Fig. 1:**
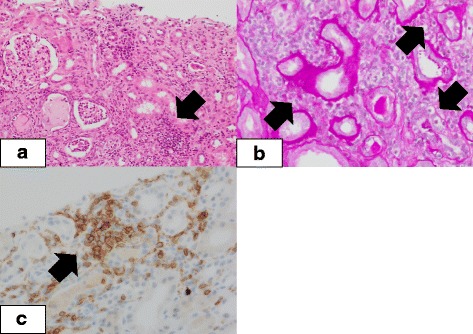
Light microscopy. **a** Hematoxylin and eosin staining. The glomeruli are mostly intact; however, marked infiltration of inflammatory cells (predominantly lymphocytes) in the interstitial space is observed (arrow). Eosinophils are not conspicuous and granuloma formation is not noted (× 200). **b** Periodic acid-Schiff staining. Lymphocyte invasion into the tubules and degeneration of tubular epithelial cells (tubulitis) are noted (arrows) (× 400). **c** Immunohistochemical study of CD4. The infiltrated cells are CD4 positive (arrow) (× 400)

**Fig. 2:**
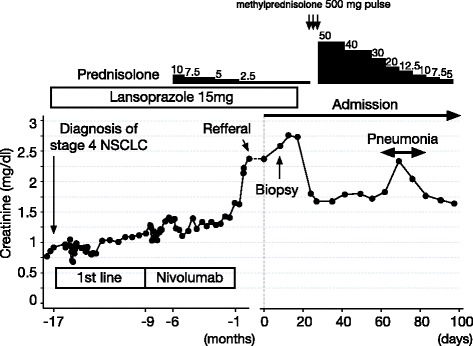
Clinical course of the patient. The patient’s creatinine levels showed rapid improvement 3 days after the discontinuation of lansoprazole, a drug for which the DLST was positive. Lansoprazole had been used safely for more than 4 years before the initiation of nivolumab therapy. DLST: drug-induced lymphocyte stimulation test; NSCLC: non-small cell lung cancer

His medications at the time of referral included aspirin 100 mg, warfarin 1.0 mg, prednisolone 2.5 mg, and lansoprazole 15 mg per day. He also used indacaterol/glycopyrronium inhaler once-daily. He had been receiving lansoprazole for more than 4 years without significant adverse effects, including kidney injury. He denied using non-steroidal anti-inflammatory drugs or other nephrotoxic agents. His weight was 49.9 kg, height was 168 cm, body temperature was 36.7 °C, pulse rate was 84/min with an irregular rhythm, and blood pressure was 102/60 mmHg. Breath sounds were reduced in the left lower lung field. His heart sounds were normal without audible murmurs. The liver, spleen, and kidneys were not palpable. Tenderness in the costovertebral angle was unclear. Skin rash, joint pain, or swelling was absent, and the oral mucosa was intact. No neurological abnormalities were noted. Laboratory investigations showed blood urea nitrogen value of 23.2 mg/dl, creatinine level of 2.39 mg/dl, and urinary beta-2 microglobulin level of 21,398 μg/l. Urine microscopy revealed numerous white blood cells (100–999 cells/high-power field) along with white blood cell casts. Blood and urine cultures were negative. Other laboratory findings are summarized in Table 1. Computed tomography (CT) revealed no possible focus of infection. A kidney biopsy was performed to clarify the cause of the acute kidney injury (AKI). The biopsied specimen contained 17 glomeruli. Two of the glomeruli were globally sclerosed but the remaining 15 showed no apparent mesangial proliferation, hypercellularity, or crescent formation. The infiltration of lymphocytes and plasma cells was conspicuous in the interstitial space and it was accompanied by mild edema and fibrosis. Tubulitis was frequently observed with mild to moderate tubular atrophy in the cortical area, but granuloma formation was not noted. Immunohistochemistry demonstrated no significant immune deposits in the glomeruli or tubules. Inflammatory cells were predominantly composed of CD4-positive cells (Fig. 1). A diagnosis of ATIN was made. As anti-PD-1 therapy could disrupt the immune tolerance to medications that had been safely used for a long time by enhancing T-lymphocyte activation, we reassessed the patient’s current medications. PPIs have been recently recognized as important offending drugs in the development of drug-induced ATIN, so lansoprazole was discontinued. His creatinine level showed rapid improvement in 3 days (decreasing from 2.74 to 1.82 mg/dl), and the sterile pyuria disappeared. The patient then received steroid therapy (500 mg intravenous methylprednisolone for 3 days followed by 1 mg/kg/day oral prednisolone), and his creatinine levels eventually stabilized around 1.7 mg/dl. DLST for lansoprazole was positive. The detailed methodology for the DLST has been described in a prior publication [8]. The patient’s clinical course is shown in Fig. 2. Since the size of the lung cancer and serum carcinoembryonic antigen level remained unchanged, the patient was followed up carefully without re-initiating nivolumab. Urinary beta-2 microglobulin level remained within normal range.

**Table 1:** Laboratory findings on admission

		Reference			Reference
Blood test					
WBC (μl)	7100	(3300–8600)	ACTH (pg/ml)	6.8	(7.2–63.3)
neutrophil (%)	75.7		cortisol (μg/dl)	17.5	(4–18.3)
lymphocyte (%)	12.0		CRP (mg/dl)	5.92	(0–0.14)
monocyte (%)	11.8		ESR (mm/h)	93	(0–9)
eosinophil (%)	0.4		ASO (IU/ml)	35	(0–166)
basophil (%)	0.1		ANA	< 40	
Hemoglobin (g/dl)	12.9	(13.7–16.8)	RF (IU/ml)	5	(0–18)
Platelet (1000/μl)	401	(158–348)	IgG (mg/dl)	1518	(861–1747)
Total protein (g/dl)	7.0	(6.6–8.1)	IgA (mg/dl)	365	(93–393)
alubmin (g/dl)	2.9	(4.1–5.1)	IgM (mg/dl)	58	(33–183)
BUN (mg/dl)	23.2	(8–18.4)	Complement 3 (mg/dl)	149	(86–160)
Creatinine (mg/dl)	2.39	(0.65–1.07)	Complement 4 (mg/dl)	56.0	(17–45)
Uric acid (mg/dl)	7.7	(3.7–7.8)	CH50 (U/ml)	71	(30–45)
Sodium (mEq/l)	142	(138–145)	C1q (μg/ml)	2.5	(0–3)
Potassium (mEq/l)	3.4	(3.6–4.8)	MPO-ANCA	(−)	
Chloride (mEq/l)	110	(101–108)	PR3-ANCA	(−)	
Calcium (mg/dl)	10.1	(8.8–10.1)	anti-GBM-ab	(−)	
iP (mg/dl)	2.5	(2.7–4.6)	cryoglobulin	(−)	
TSH μIU/ml)	0.32	(0.5–5)	PCT (ng/ml)	0.108	(0–0.5)
fT3 (pg/ml)	1.88	(2.3–4)	Blood culture	(−)	
fT4 (ng/dl)	1.17	(0.9–1.7)			
Urinalysis					
pH	6.0		RBC casts (/HPF)	1–4	
Glucose	(−)		WBC casts (/HPF)	> 100	
Ketones	(−)		β2-microglobulin (μg/l)	21,398	(13–287)
Blood	(1+)		NAG (U/l)	13.7	(< 11.3)
Protein	(1+)		Protein excretion (g/day)	0.204	(< 0.15)
DLST					
lansoprazole (S.I)	3.6	(< 1.6)			

*ACTH* adrenocorticotropic hormone, *ANA* anti-nuclear antibody, *ANCA* anti-neutrophil cytoplasmic antibody, *ASO* anti-streptolysin O antibody, *BUN* blood urea nitrogen, *CH50* 50% hemolytic complement activity, *CRP* C-reactive protein, *DLST* drug induced lymphocyte stimulating test, *ESR* erythrocyte sedimentation rate, *GBM* anti-glomerular basement membrane antibody, *MPO* myeloperoxidase, *NAG* N-acetyl-β-D-glucosaminidase, *PCT* procalcitonin, *PR3* proteinase3, *RF* Rheumatoid Factor, *S.I* stimulation index, *TSH* thyroid stimulating hormone

## Discussion and conclusions

In the largest case series that evaluated ICPI-associated AKI, 12 out of 13 patients had ATIN [5]. The reasons why ATIN is the common pathological feature of ICPI-associated AKI remain unknown. The causes of ATIN include drugs, infections, and autoimmune diseases such as Sjögren’s syndrome, sarcoidosis, systemic lupus erythematosus, and IgG4-related kidney disease. Although the precise pathophysiology of drug-induced ATIN is unknown, drug-specific T-cells have been shown to play a crucial role [7]. However, PD-1 knockout mice have shown the autoimmune disease-like phenotype and development of glomerulonephritis, revealing the critical role of PD-1 signaling in the regulation of self-immunological tolerance [9]. Considering these facts, it is plausible that the blockade of the PD-1 signaling pathway by ICPI therapy can result in ATIN by altering T-cell immune tolerance against concomitant drugs (drug-induced) or kidney intrinsic antigens (autoimmune related). The detailed mechanism underlying the induction of ATIN due to the loss of T-cell immune tolerance is yet to be elucidated. It has been reported that some auto-reactive T-cells escape negative selection in the thymus and are kept dormant by several mechanisms to prevent autoimmunity [10]. PD1/PD-L1 signals contribute to maintaining peripheral T-cell immune tolerance [11]. Renal tubular cells express PD-L1, which protects them from T-cell-mediated autoimmunity [12]. Murakami et al. proposed an intriguing hypothesis that re-activation of these dormant auto-reactive T-cells by immune checkpoint inhibitors can lead to organ-specific injury [13]. Thus, anti-PD-1 antibody treatment can disrupt the peripheral immune tolerance between renal tubular cells and dormant auto-reactive T-cells. This hypothesis seems reasonable because it explains the development of tubulointerstitial nephritis after PD-1 therapy irrespective of whether re-activated T-cells recognize specific drugs or kidney intrinsic antigens.

Because of their safety and efficacy, PPIs have been used globally for the treatment of acid-related gastroesophageal disease. However, a nationwide nested case-control study demonstrated that the use of PPIs increases the risk of ATIN [14]. In a case series from a single center that included 133 patients with biopsy-proven ATIN, 13 of 95 drug-induced ATIN cases (11%) were induced by PPIs [15]. Recently, some epidemiological studies reported the relationship between PPI use and the prevalence of chronic kidney disease [16], suggesting the contribution of clinically unrecognized drug-induced ATIN. PPIs can trigger host immune response by haptenization (acquiring immunogenicity by binding larger host proteins) or direct interaction with immune receptors or major histocompatibility complex (MHC) proteins (pharmacologic interaction) [17]. Shirali et al. reported 6 cases with biopsy-proven ATIN during treatment with nivolumab or pembrolizumab in lung cancer patients [6]. Five out of 6 cases in their report received PPIs. Among them, kidney function improved in 4 cases after discontinuation of PPI and steroid therapy, but 2 cases experienced a relapse of kidney injury with the occasional use of PPI. Moreover, they reported one case in which ATIN (peak creatinine level 1.9 mg/dl) was managed with the discontinuation of PPI (omeprazole) therapy alone, without steroid therapy. Notably, in this case, nivolumab therapy was continued for 8 months after discontinuation of omeprazole (creatinine level fluctuated from 1.4 to 1.7 mg/dl). They speculated that the blockade of PD-1 signaling could lead to the development of ATIN by modulating peripheral immune tolerance for the drugs that had been safely used previously. These facts informed our decision to discontinue lansoprazole, and we observed rapid improvement in the patient’s kidney function after discontinuing the drug.

Although it is possible that ATIN was caused by other etiologies in this patient, infection was not suspected since physical findings were unremarkable, blood and urine culture were negative, and radiological survey including CT scanning identified no apparent focus, and the procalcitonin level was not elevated. The development of autoimmune disease-like symptoms is being increasingly recognized during ICPI treatment. Lupus nephritis was reported in a man with advanced-stage melanoma during treatment with the anti-CTLA-4 antibody ipilimumab [18], but the present case showed no typical manifestations or serological abnormalities suggesting the complication of autoimmune disease that could cause ATIN. The rapid improvement of kidney function after the discontinuation of lansoprazole and DLST positivity with lansoprazole strongly indicate that this drug played a principal role in the development of ATIN in this patient, who had received this drug uneventfully for more than 4 years prior to this incident. PPI-induced ATIN has been reported to occur within 10 days to 18 months after drug initiation [19]. Considering the time course, the blockade of the intrinsic PD-1 signaling pathway with anti-PD-1 therapy possibly disrupted the patients’ long-standing immunological tolerance against lansoprazole by modulating T-cell immunoreactivity. To the best of our knowledge, this is the first case report that confirmed DLST positivity against a drug that had been used safely for a long time until the initiation of anti-PD-1 therapy. It is possible that nivolumab therapy affects the result of in vitro tests, such as the DLST. However, considering the half-life of nivolumab in the human body (12 to 20 days) [20] and the time period between nivolumab discontinuation and the day on which the DLST was performed (70 days), the patient’s serum nivolumab concentration would have been very low at the time of the DLST test. Moreover, an experimental study demonstrated that nivolumab did not evoke nonspecific T-cell activation in vitro [21]. Considering all these findings, we believe that our patient’s DLST result reflects the T-cell response against lansoprazole.

The histological findings of interstitial fibrosis and tubular atrophy may explain the fact that the patient’s kidney function did not return to the baseline (1.2 mg/dl) after steroid therapy. Although discontinuation of the culprit drug alone resulted in a significant improvement in kidney function, the response to subsequent steroid therapy was not as strong as expected (from 1.82 mg/dl to approximately 1.7 mg/dl). In fact, there is no conclusive evidence of the efficacy of steroid treatment for drug-induced tubulointerstitial nephritis. However, a recent study demonstrated that early steroid treatment is beneficial for the recovery of kidney function in patients with drug-induced ATIN [22]. The study proposed that early steroid treatment can prevent the progression of interstitial fibrosis that occurs soon after discontinuation of the culprit drug. Thus, steroid therapy might have prevented the progression of interstitial fibrosis after the discontinuation of lansoprazole in this patient. In addition, we cannot deny the possibility that steroid therapy also suppressed the patient’s auto-reactive T-cells against kidney intrinsic antigens that were previously kept dormant by host PD-1 signals. Based on these findings, we suggest that in addition to discontinuation of the culprit drug, early steroid therapy might be beneficial for patients with ICPI-induced ATIN in whom the concomitant causative agent is identified in order to prevent the progression of interstitial fibrosis (and to suppress the possible action of auto-reactive T-cells against kidney intrinsic antigens).

Our study has several limitations. First, the DLST for lansoprazole was not performed before nivolumab therapy; thus, it is not known whether nivolumab therapy actually altered the patient’s immunological tolerance against lansoprazole. Second, the sensitivity and specificity of the DLST in a PPI-induced ATIN setting have not yet been determined; therefore, the positive DLST result does not necessarily indicate a causal relationship between lansoprazole usage, nivolumab treatment, and ATIN development. Third, the direct role of nivolumab as a causative agent of ATIN remains uncertain because the DLST against nivolumab itself was not performed for this patient. Further case reports including DLST results for suspicious drugs are required to clarify the pathophysiology of ATIN and to establish the practical management of AKI during ICPIs therapy. ICPIs can be re-challenged without renal irAEs relapse after steroid therapy for ATIN [5, 23]. However, except for a few case series [5, 6], detailed information about the timing for the discontinuation or re-initiation of concomitant drugs during the clinical course is not available in recent case reports. Thus, it is unclear whether recovery of kidney function (and uneventful re-initiation of ICPI therapy in some cases) is attributable to the steroid therapy or the discontinuation of the culprit drug in these cases. As the patients receiving ICPI therapy have advanced stage cancer, the risks and benefits of re-initiating ICPIs need to be meticulously considered. Our case indicates the importance of recognizing concomitant medications known to cause ATIN during the treatment of AKI associated with PD-1 blockade. The identification and discontinuation of such drugs may prevent the relapse of kidney injury and enable safe re-initiation of ICPI therapy.

## Abbreviations

AKI: Acute kidney injury
ATIN: Acute tubulointerstitial nephritis
CTLA-4: Cytotoxic T-lymphocyte antigen 4
DLST: Drug-induced lymphocyte stimulation test
ICPI: Immune checkpoint inhibitor
irAEs: Immune-related adverse events
PD-1: Programmed cell death protein 1
PD-L1: Programmed cell death protein 1 ligand
PPI: Proton pump inhibitor
